# Inadequate vancomycin therapy in term and preterm neonates: a retrospective analysis of trough serum concentrations in relation to minimal inhibitory concentrations

**DOI:** 10.1186/1471-2431-14-193

**Published:** 2014-07-28

**Authors:** Fleur S Sinkeler, Timo R de Haan, Caspar J Hodiamont, Yuma A Bijleveld, Dasja Pajkrt, Ron A A Mathôt

**Affiliations:** 1 Departments of Hospital Pharmacy – Clinical Pharmacology, Amsterdam, The Netherlands; 2 Neonatology, Emma Children’s Hospital, Amsterdam, The Netherlands; 3 Medical Microbiology, Amsterdam, The Netherlands; 4 Pediatric Infectious Diseases, Emma Children’s Hospital, Academic Medical Centre, Amsterdam, The Netherlands; 5 Academic Medical Center – Department of Clinical Pharmacy, PO Box 22660, 1100 DD Amsterdam, The Netherlands

**Keywords:** MIC-values, Neonatal, Therapeutic Drug Monitoring, Vancomycin, Staphylococcal sepsis

## Abstract

**Background:**

Vancomycin is effective against gram-positive bacteria and the first-line antibiotic for treatment of proven coagulase-negative staphylococcal infections. The aim of this study is bipartite: first, to assess the percentage of therapeutic initial trough serum concentrations and second, to evaluate the adequacy of the therapeutic range in interrelationship with the observed MIC-values in neonates.

**Methods:**

In this study, preterm and term neonates admitted at a tertiary NICU in the Netherlands from January 2009 to December 2012 and treated with vancomycin for a proven gram-positive infection were included. Trough serum concentrations were measured prior to administration of the 5th dose. Trough concentrations in the range of 10 to 15 mg/L were considered therapeutic. Staphylococcal species minimal inhibitory concentrations (MIC’s) were determined using the E-test method. Species identification was performed by matrix-assisted laser desorption/ionisation mass spectrometry.

**Results:**

Of the 112 neonates, 53 neonates (47%) had sub-therapeutic initial trough serum concentrations of vancomycin, whereas 22% had supra-therapeutic initial trough serum concentrations. In all patients doses were adjusted on basis of the initial trough concentration. In 40% (23/57) of the neonates the second trough concentration remained sub-therapeutic. MIC’s were determined for 30 coagulase-negative *Staphylococcus* isolates obtained from 19 patients. Only 4 out of 19 subjects had a trough concentration greater than tenfold the MIC.

**Conclusions:**

Forty-seven percent of the neonates had sub-therapeutic initial trough serum concentrations of vancomycin. The MIC-data indicate that the percentages of underdosed patients may be greater. It may be advisable to increase the lower limit of the therapeutic range for European neonates.

## Background

Ongoing improvement in the care of increasingly younger and critically ill neonates in Neonatal Intensive Care Units (NICU) coincides with an increased use of indwelling central venous catheters (CVC). Neonates are highly susceptible to invasive gram-positive infections associated with use of these CVCs. Coagulase-negative *Staphylococcus* (CNS) and *Staphylococcus aureus* are the most common causative pathogens found in neonatal central line sepsis [1-3]. Vancomycin is an effective agent against gram-positive bacteria, especially resistant staphylococci, and is therefore the first line antibiotic for treatment of proven coagulase-negative staphylococcal infections [4,5].

Adequate dosing of vancomycin is of major importance as too low or too high plasma concentrations can lead to ineffective therapy or toxicity, respectively. As a result, therapeutic drug monitoring (TDM) is applied in order to optimize vancomycin therapy. Several vancomycin dosage regimens have been proposed for term and preterm neonates. Most of these dosage regimens are based on postconceptional age only [6]. Other studies suggest regimens based on serum creatinin only or regimens irrespective of gestational or postconceptional age [7,8].

An efficacy parameter for adequate vancomycin therapy is currently missing and needed. The ratio of the area under the serum concentration versus time curve and the minimal inhibitory concentration (AUC/MIC) has been suggested as an efficacy parameter. The AUC/MIC, derived from the pharmacokinetic profile of the patient, can be used to rationalise dosage regimens for individual neonatal patients [9]. This option has however not yet been investigated in the neonatal population. For monitoring purposes assessment of vancomycin trough concentrations is more feasible. A vancomycin trough serum concentration of tenfold the MIC is generally considered the lower limit for the therapeutic concentration range. It has been reported that an AUC/MIC of approximately 400 corresponds to a trough serum concentration of 8 to 9 mg/L [10].

This study was designed to answer two clinical questions. First, to assess the percentage of sub-therapeutic trough serum concentrations in a cohort of both term and preterm neonates and second, to evaluate the adequacy of the currently used therapeutic range in relationship with the observed MIC-values.

## Methods

The study population consisted of a cohort of preterm and term neonates admitted at the tertiary NICU of the Academic Medical Centre in Amsterdam, The Netherlands, from January 2009 to December 2012, who were treated with vancomycin for a proven gram-positive infection. Patient characteristics were extracted from the clinical and microbiological database. All neonatal patients receiving vancomycin therapy were eligible for study inclusion, regardless of gestational age or birth weight. Exclusion criterion was either an unavailable vancomycin trough concentration or no or insufficient documentation on dosage schedules.

Due to the retrospective nature of this study and because the subjects and data were unidentifiable from the reported analyses and data, the institutional medical ethical committee of the Academic Medical Centre (Amsterdam, The Netherlands) decided that approval and informed consent were not needed for this study, which is in line with the Dutch Medical Research (Human Subjects) Act.

Vancomycin was administered intravenously and dosed according to hospital dosing guidelines based on national pediatric dosing guidelines [11]. The applied dosage regimen is shown in Table 1. Trough blood samples were taken routinely before administration of the fifth vancomycin dose according to hospital protocol. Trough serum concentrations in the range of 10 to 15 mg/L were considered therapeutic. The total numbers (and%) of cases with trough concentrations below and above the therapeutic range (10 – 15 mg/L) were assessed.

**Table 1:** Vancomycin dosing regimen

**Age categories**		**Daily dose**	**Dose frequency**
		**(mg/kg/day)**	**(dose per 24 h)**
**I**	< 26 weeks PMA	15	1 dose
**II**	26 – 37 weeks GA & < 7 days PNA	20	2 doses
**III**	> 37 weeks GA & < 7 days PNA	30	2 doses
**IV**	PNA > 7 days (preterm and term)	40	2 doses

Legend: GA: gestational age, PMA: postmenstrual age, PNA: postnatal age.

The following patient characteristics were obtained from the medical records: gender, gestational age (in weeks), birth weight (in grams), postnatal age (in days) at start of vancomycin treatment, weight (in grams) at the time of vancomycin treatment, duration of treatment (in days), relevant co-medication possibly influencing drug clearance (ibuprofen or indometacin).

Vancomycin susceptibility was tested for all coagulase-negative staphylococci isolated from bloodcultures of infants receiving vancomycin between January and December 2012. Vancomycin minimal inhibitory concentrations (MIC’s) were determined using the E-test method according to the manufacturer’s guidelines (Biomérieux, Marcy l’Etoile, France). If more than one isolate was cultured from the same patient during the same episode, subsequent isolates of the same species were excluded if they showed an identical susceptibility pattern. Species identification was performed by matrix-assisted laser desorption/ionisation mass spectrometry, using MALDI Biotyper software v3 (Bruker Daltonik GmbH, Bremen, Germany).

SPSS version 18.0 was used to analyze the data and describe patient demographics. Bivariate correlation was investigated between measured trough levels and patient characteristics. As data were not normally distributed, Spearman’s rho was calculated.

## Results

**Figure 1:**
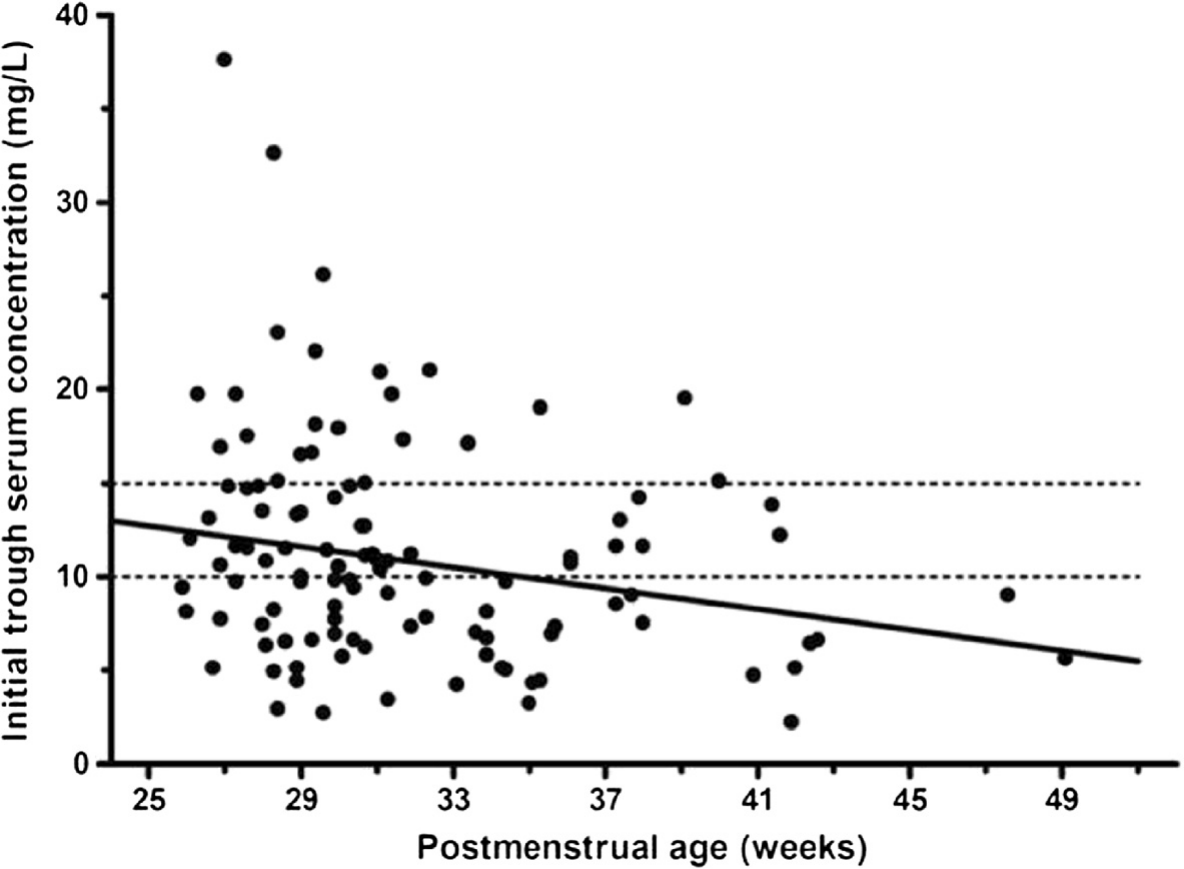
**Initial vancomycin serum trough concentration vs postmenstrual age.** Initial vancomycin serum trough concentration versus postmenstrual age for 112 neonates. The dashed lines indicate the therapeutic range of vancomycin trough concentration (10 – 15 mg/L). The solid line was obtained by linear regression analysis (Spearman’s rho): r = -0.248, p = 0.009.

A total of 116 neonates were included in this study. Four neonates lacked appropriately documented serum concentrations and were therefore excluded from the study. A remaining total of 112 initial trough serum concentrations were analyzed. The first patient category (postmenstrual age (PMA) < 26 weeks) included only 1 patient, the second category (gestational age (GA) 26 – 37 & postnatal age (PNA) < 7 days) 2 patients, the third category (GA > 37 weeks & PNA < 7 days) 4 patients and the last category (preterm or term, PNA > 7 days) included 105 patients. Subjects in the fourth and most substantial age category demonstrated a median gestational age of 28 weeks (range 24 – 41 weeks), a median birth weight of 890 grams (range 430 – 4140 grams), a median postnatal age at start of the therapy of 14 days (range 3 – 112 days), and median weight at start of the therapy of 1040 grams (range 500 – 4310 grams).Combining all groups, the median initial trough serum concentration was 10.6 mg/L (range 2.2 – 37.6 mg/L, n = 112). In the fourth group, the median initial trough serum concentration was 10.5 mg/L (range 2.7 – 37.6 mg/L, n = 105). In all groups, a total of 53 of 112 patients (47%) had a sub-therapeutic (<10 mg/L) initial trough serum concentration of vancomycin, whereas 22 of 112 patients (20%) had a supra-therapeutic (>15 mg/L) initial trough serum concentration (Figure 1).

TDM was performed in all of the 112 patients; doses were adjusted in order to obtain trough concentrations in the therapeutic range. During continued therapy a second trough serum concentration was determined in 57 of 112 patients. Vancomycin therapy was stopped shortly (within 3 days) after the initial trough serum concentration in the remaining 55 subjects for varying reasons (mostly after removal of the infected CVC). In 52 out of 57 patients with a second trough serum concentration the vancomycin dosage was adjusted. Twenty-three of the 57 neonates (40%) still demonstrated sub-therapeutic trough concentrations after dose adjustment. Dose adjustment resulted in a median trough serum concentration of 11.0 mg/L (range 3.1 – 37.7 mg/L, n = 57).In the studied population initial trough serum concentrations were significantly correlated with the gestational age (r = -0.250, p = 0.03) and the postmenstrual age at the start of the therapy (r = -0.248, p = 0.009). Figure 1 shows the relationship between the initial trough serum concentrations and PMA of the entire population.

In total, 30 coagulase-negative *Staphylococcus* isolates from 19 patients were tested for MIC. Staphylococcal isolates consisted of 19 *S. epidermidis*, 9 *S. capitis* and 2 *S. warneri* isolates. Of these 30 isolates, 2 (7%) showed an MIC ≤ 0.5 mg/L, 9 (30%) showed an MIC of 1.5 mg/L, 17 (58%) showed an MIC of 2.0 mg/L and 2 (7%) showed an MIC of 3.0 mg/L. Tenfold the value of the MIC is considered the lower limit for therapeutic trough serum concentrations. By this definition, only 4 out of 19 (21%) patients (where the MIC was tested) reached therapeutic trough concentrations. Two patients had an isolate with a MIC ≤ 0.5 mg/L, 1 patient showed an isolate with a MIC of 1.5 mg/L and 1 patient showed an isolate with a MIC of 2.0. These patients achieved a therapeutic concentration tenfold the MIC. All were classified in the fourth age category.

Follow-up blood cultures were available in 14 of the 19 subjects. In 13 of the 14 subjects, follow-up blood cultures were negative, in follow-up blood cultures of 1 subject the initial CNS species was not found, but a new micro-organism was isolated.

The mortality in the present study cohort was 18% (21/112). Of these 21 subjects, 6 neonates died due to respiratory and circulatory compromise during an episode of severe clinical sepsis. In 4 of these 6 subjects, CNS was isolated, while in 2 subjects the causative micro-organism was not found. In the sub-therapeutic group (mortality 7/21), 3 subjects died due to complications of severe necrotizing enterocolitis (NEC): in 1 patient intensive care treatment was withdrawn following severe perinatal asphyxia and a large cerebral hemorrhage caused demise in 1 other infant. Ongoing neonatal sepsis was the cause of death in 2 other subjects. Both of these suffered ongoing CNS sepsis, one in the presence of a large vena cava inferior thrombus.

In the supra-therapeutic group (mortality 4/21) 1 subject died because of respiratory failure in severe bronchopulmonary dysplasia (BPD). Three other subjects died due to complications of clinical sepsis. One of these suffered sepsis due to a combined *Enterobacter* species and *S. epidermis infection*.

In the therapeutic group (mortality 10/21), 5 patients died as a result of NEC, 2 due to multi-organ failure following perinatal asphyxia and 2 due to severe respiratory and circulatory insufficiency (primary pulmonary hypertension of the neonate, PPHN). One patient died as a result of an ongoing CNS infection.

## Discussion

In the present study, we studied initial trough serum concentrations of vancomycin in critically ill neonates. In this study, only about one third of all subjects had adequate initial trough serum concentrations, indicating inadequate dosing in the majority of subjects, thus exposing a large number of subjects to either the risk of ongoing infection or complications due to vancomycin toxicity.

There is significant discussion regarding the adequate range of trough concentrations for successful treatment. Therapeutic trough vancomycin concentrations range from 5 mg/L to 25 mg/L [8,12]. Thus, reports on the number of subjects with sub-therapeutic levels of vancomycin show wildly varying results, with reported percentages ranging from 24% to 58% [13,14]. In the present study, approximately half of the neonates had initial trough serum concentrations in the sub-therapeutic range after the administration of the recommended dosing regimen. Unexpectedly, after TDM, the percentage of patients with sub-therapeutic serum concentrations decreased only from an initial 58% to 40% of patients. This moderate increase in the number of subjects reaching therapeutic serum concentrations may be hampered by the significant increase of renal function shortly after birth. As a result vancomycin clearance rates may increase considerably during a short period of time. Dose adjustments based on initial trough serum concentrations should therefore also take the maturation of renal function into account.

Conversely, 8 subjects had supra-therapeutic levels of vancomycin. Most of these subjects were under 33 weeks of gestational age. In these very young subjects there is a potential for nephrotoxicity. Unfortunately, our study could not provide any evidence of nephrotoxicity as follow-up of serum creatinine was not available in most subjects. However, the rapid increase in renal function shortly after birth, may significantly help overcome any supra-therapeutic levels of vancomycin and hence reduce the risk of toxicity.

Previous studies have identified age, weight and renal function as determinants for vancomycin concentrations [6,12]. In this study, we confirmed the association between gestational age and initial trough serum concentrations of vancomycin. Although our method to determine correlations between patient differences was basic and more accurate methods to determine correlations are available, the influence of age on serum concentrations was statistically significant, but small. It is remarkable that an association between age and serum concentrations was still detected, since the currently used dosing schemes should take the age dependency of clearance into account. These results therefore suggest the current dosing regimen may not be sufficient and should be modified. We were unable to demonstrate any significant correlations between initial trough serum concentrations and weight at start of the therapy, contrary to results of other studies [12].

Weight and age are strongly correlated in this population, which partly explains the relation of weight and initial trough serum concentrations in other studies. At least, our results suggest dosing based solely on weight may not be adequate to achieve therapeutic levels in neonates.

It is important to note that the clinical efficacy of vancomycin in neonates is not only determined by the trough serum concentration and factors influencing clearance rates. Clinical efficacy, and thus the therapeutic range, depends on the MIC of the involved micro-organism(s), and this may vary among centers or countries. Reports on therapeutic ranges in one center may not necessarily reflect therapeutic ranges from another center. There is a need for individualized therapy, based on individual MIC-values.

To the best of our knowledge, this is the first study to correlate MIC-values with initial trough serum concentrations. Only 21% of the subjects reached an initial trough concentration above tenfold the MIC, thus the vast majority of subjects may have received inadequate therapy. The best predictor of a clinical outcome of vancomycin therapy is the AUC/MIC ratio, with an AUC/MIC value for clinical effect greater than 400 [15]. In clinical practice, it is unfeasible to obtain an AUC, since serial plasma concentrations are needed for calculation of the AUC. The trough serum concentrations may be used as another parameter to reflect drug exposure [16]. The causative CNS spp. in our patients showed an MIC ≥ 1.5, which is comparable to CNS MIC data from the European EUCAST reference database, where 93% of coagulase-negative *Staphylococci* showed an MIC in the range of 1.0 to 2.0 mg/L [17]. This demonstrates that our CNS spp. can probably be compared to spp. detected in the surrounding European countries.

Although the use of the E-test may overestimate MIC-values compared to broth microdilution (as used by EUCAST) [18,19], it does appear to be more reliable in predicting treatment response compared to the EUCAST method [18]. In light of these considerations, our data may be extrapolated to other comparable European populations.

Based on the MIC findings in our population and the MIC-values of the European EUCAST, increasing the range of the therapeutic trough concentration for vancomycin to 15 – 20 mg/L might well be advisable, not only in our hospital, but in other centers as well.

In total, 21 out of 112 subjects died, of whom three subjects died due to an ongoing staphylococcal infection. These subjects were equally distributed over the sub-, supra- and therapeutic group. However, due to lack of information on concurrent morbidities and lack of MIC, it is impossible to draw conclusions on the association between sub-therapeutic serum concentrations and mortality in this study. In the first three age categories, 3 out of 7 patients had sub-therapeutic initial trough serum concentrations, whereas 1 patient had a supra-therapeutic initial trough serum concentration. Almost all our patients were included in the fourth age category, as treatment of infections with vancomycin in subjects under 7 days of age is not considered a standard treatment. Thus, considering the small number of patients in the first three age categories, our study results can only be applied to patients with a postnatal age > 7 days, a median gestational age of 28 weeks (range 24 – 41 weeks) and with a median birth weight of 890 grams (range 430 – 4140 grams). This is however the most crucial neonatal class of patients admitted to a NICU and prone to suffering a central line sepsis.

## Conclusions

In conclusion, we found that the vast majority (47%) of neonates treated with vancomycin had sub-therapeutic initial trough serum concentrations. Furthermore, the observed MIC values indicate that the percentage of underdosed patients may even be greater. Based on the MIC data, it may be advisable to raise the lower limit of the therapeutic range of vancomycin to 15 mg/L for European neonates with a postnatal age > 7 days, a birth weight < 1000 grams and gestational age < 28 weeks. While increasing the therapeutic range will be most certainly increase the efficacy of vancomycin for the treatment of gram-positive bacteremia, this may also give rise to a potential increase in toxicity and adverse effects of vancomycin. Future well-designed (preferably multi-center) prospective pharmacokinetic and –dynamic studies should evaluate the association between dosing schedules, micro-organism profiles and therapy efficacy, preferably including long term patient outcome. It would be of patient interest to apply our results by adjusting the current dosing scheme to allow for more adequate initial trough serum concentrations in these fragile patients, at high risk for adverse outcomes.

## Abbreviations

AUC: Area under the concentration versus time curve; BPD: Bronchopulmonary dysplasia; CNS: Coagulase-negative Staphylococcus; CVC: Central venous catheters; EUCAST: European committee on antimicrobial susceptibility testing; GA: Gestational age; MIC: Minimal inhibitory concentrations; NEC: Necrotizing enterocolitis; NICU: Neontal intensive care unit; PMA: Postmenstrual age; PNA: Postnatal age; PPHN: Primary pulmonary hypertension of the neonate; TDM: Therapeutic drug monitoring.

## Competing interests

The authors declare that they have no competing interests.

## Authors’ contributions

FSS drafted the manuscript and collected the data, TRH helped with collecting, analysis and interpretation of the data and helped to draft the manuscript, CJH tested blood cultures and determined MIC’s, YB collected the data, DP helped with analysis of the data, RAAM helped with analysis and interpretation of the data and helped to draft the manuscript. All authors have revised the manuscript and read and approved the final manuscript.

## Pre-publication history

The pre-publication history for this paper can be accessed here:

http://www.biomedcentral.com/1471-2431/14/193/prepub
